# Identification of arginine- and lysine-methylation in the proteome of *Saccharomyces cerevisiae *and its functional implications

**DOI:** 10.1186/1471-2164-11-92

**Published:** 2010-02-05

**Authors:** Chi Nam Ignatius Pang, Elisabeth Gasteiger, Marc R Wilkins

**Affiliations:** 1 School of Biotechnology and Biomolecular Sciences, University of New South Wales, Sydney, NSW, 2052, Australia; 2 Systems Biology Initiative, University of New South Wales, Sydney, NSW, 2052, Australia; 3 Swiss Institute of Bioinformatics, Swiss-Prot Group, CMU - 1, rue Michel Servet, CH-1211 Geneva 4, Switzerland

## Abstract

**Background:**

The methylation of eukaryotic proteins has been proposed to be widespread, but this has not been conclusively shown to date. In this study, we examined 36,854 previously generated peptide mass spectra from 2,607 *Saccharomyces cerevisiae *proteins for the presence of arginine and lysine methylation. This was done using the FindMod tool and 5 filters that took advantage of the high number of replicate analysis per protein and the presence of overlapping peptides.

**Results:**

A total of 83 high-confidence lysine and arginine methylation sites were found in 66 proteins. Motif analysis revealed many methylated sites were associated with M**K**, **R**GG/**R**XG/**R**GX or WXXX**R **motifs. Functionally, methylated proteins were significantly enriched for protein translation, ribosomal biogenesis and assembly and organellar organisation and were predominantly found in the cytoplasm and ribosome. Intriguingly, methylated proteins were seen to have significantly longer half-life than proteins for which no methylation was found. Some 43% of methylated lysine sites were predicted to be amenable to ubiquitination, suggesting methyl-lysine might block the action of ubiquitin ligase.

**Conclusions:**

This study suggests protein methylation to be quite widespread, albeit associated with specific functions. Large-scale tandem mass spectroscopy analyses will help to further confirm the modifications reported here.

## Background

The methylation of proteins is of increasing biological interest. It is predominantly found on lysine and arginine residues, but has also been found on histidine, glutamic acid and on the carboxyl groups of proteins (reviewed in Grillo and Colombatto 2005) [1]. Methylation of lysine involves the addition of one to three methyl groups on the amino acid's ε-amine group, to form mono-, di- or tri-methyllysine. Its function is best understood in histones. Methylation on the tails of histone proteins, in conjunction with acetylation and phosphorylation, controls their interaction with other proteins, affects chromatin compaction and the up- or down-regulation of gene expression [2]. For *S. cerevisiae*, lysine methylation is found in histone H3 and histone H4 [3]. Tri-methylation at H3K4 and H3K36 is positively correlated with gene activity [4], while H3K79 are involved in gene silencing [5,6]. Histone H3K79 methylation is evolutionarily conserved and is involved in several pathways, including Sir protein-mediated heterochromatic gene silencing [7]. meiotic checkpoint control [8] and in the G1 and S phase DNA damage checkpoint functions of Rad9p [9,10]. While studies of lysine methylation have mainly focused on histone proteins, several non-histone proteins are also known to be lysine-methylated. They are mainly ribosomal proteins or proteins involved in protein translation [11], and include Rpl12p [12,13], Rpl23p [12,14], Rpl42p [15], and eEF1Ap [16].

The methylation of arginine involves the addition of one or two methyl groups to the amino acid's guanidino group, forming mono- or di-methylarginine. It is predominantly known to be associated with RNA regulation and processing [17]. In *S. cerevisiae*, Hmt1p is a type 1 arginine methyltransferase that catalyses the formation of mono- and asymmetric di-methylarginine. This enzyme is known to methylate a number of proteins that contain an RGG-motif; these include Npl3p, Hrp1p, Nab2p, Gar1p, Nop1p, Nsr1p, Yra1p, Sbp1p, and Hrb1p. These proteins have been implicated in poly(A)+ mRNA binding, processing and export [17], ribosome biogenesis [18-20] and gene silencing [21]. Moreover, methylation is required for the nuclear export of RNA binding proteins Npl3p, Hrp1p, and Nab2p [22,23]. The repeated RGG-motif was known as a RNA-binding motif [24], and this also supports the role of arginine methylation in the regulation of mRNA binding [25]. The methylation of nuclear shuttling proteins is suggested to weaken their binding with cargo proteins and disrupt their export from the nucleus [26]. Arginine methylation is also known to facilitate or block protein-protein interactions. Arginine methylation of SmB protein facilitates the binding of tudor domains in SMN, SPF30, and TDRD3 proteins [27]. In contrast, arginine methylation of Sam68 blocks the interaction of nearby proline-rich motif with an SH3 domain, but not to a WW domain [28]. More examples on methylarginine-regulated interactions are reviewed in McBride and Silver (2001) [29] and Bedford and Clarke (2009) [30].

There have been several studies to identify arginine or lysine-methylated proteins on a proteome-wide scale. In the first of these studies, arginine-methylated protein complexes were purified from HeLa cell extracts using anti-methylarginine antibodies specific against RG-rich sequences [31]. This resulted in the identification of over 200 arginine-methylated proteins, involved in pre-mRNA processing, protein translation, and DNA transcription. However the actual methylation sites on these proteins remain unknown [32]. The second study utilised stable isotope labelling by amino acid in cell culture (SILAC), in which [^13^CD_3_]methionine was converted to [^13^CD_3_]S-adenosyl methionine, the substrate for arginine and lysine methylation [32]. Advantages of this method included increased confidence of identification, a capacity to distinguish between trimethylation and acetylation which are near-isobaric, and the ability to quantify the relative changes in methylation status of a protein between two samples. In combination with anti-methyllysine and anti-methylarginine antibody immunoprecipitation techniques, Ong *et al. *(2004) [32] was able to identify methylation on histones from HeLa cell extracts, such as on histone H3K27. Around 30 other proteins were also found to be methylated at RG-rich motifs and most of these proteins are RNA binding or associated with mRNA processing pathways. The third study used anti-methyllysine antibodies to search for organ-specific lysine methylation in *Mus musculus *[33]. Proteomic analysis of brain tissue extract by 2-D PAGE, western blotting, and MALDI-ToF peptide mass fingerprinting identified the following lysine-methylated proteins: neurofilament triplet-I protein, Hsc70 protein, creatine kinase, α-tubulin, α-actin, β-actin, and γ-actin. Furthermore, α-actin and creatine kinase were found to be methylated in muscle tissue.

The use of tandem mass spectrometry to discover new protein post-translational modifications is common [34]. However, peptide mass fingerprinting can also be used to search for new PTM sites [35]. The FindMod program [35] caters for this approach. It requires peptide mass spectra from a mostly pure protein, for example a spot from 2-D gel, and examines experimental peptide masses for differences in mass with theoretical peptides for that protein that correspond to post-translational modification. Peptides that are potentially modified are checked to see if they contain amino acids that can carry the modification. Where very high accuracy peptide mass measurements can be made, for example with new instruments like the prOTOF2000, high confidence predictions are possible. Parent-ion masses from tandem mass spectrometry data can also be used in FindMod, where it may serve as an initial screen for PTMs before employing more sophisticated and computationally expensive methods [36,37].

Here we describe a strategy for the discovery of methylation on a global scale, using peptide mass fingerprinting data, and implement this to search for methylated lysine and arginine residues in the yeast proteome. A proteome-scale set of MALDI-ToF mass spectra [38] was analysed for putative methylated peptides. The application of 5 filters yielded high-confidence methylation sites that were then further investigated to understand where they are found in protein sequences and their likely function.

## Results

### Large-scale methylation discovery in yeast peptide mass spectra

**Figure 1:**
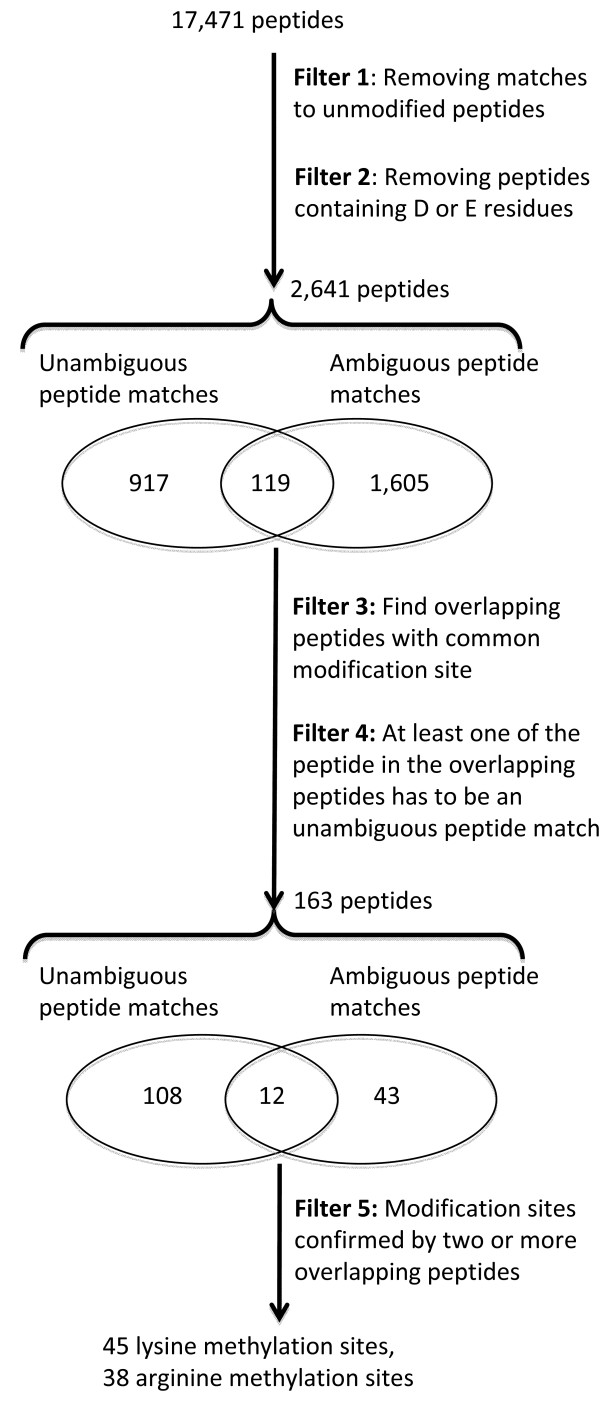
**Finding high confidence modified peptides using 5 filtering strategies**. By removing peptides that matched to both methylated peptides and unmodified peptides (filter 1) and peptides which contains D or E residues (filter 2), 2,641 peptides were left. By filtering for overlapping peptides (filter 3), and where at least one of the overlapping peptide is an unambiguous peptide match (filter 4), 163 peptides remained. These included 108 unambiguous peptide matches, 43 ambiguous peptide matches, and 12 peptides in both categories. Found in these peptides are 45 lysine methylation sites and 38 arginine methylation sites, all of which are of high confidence (filter 5).

**Figure 2:**
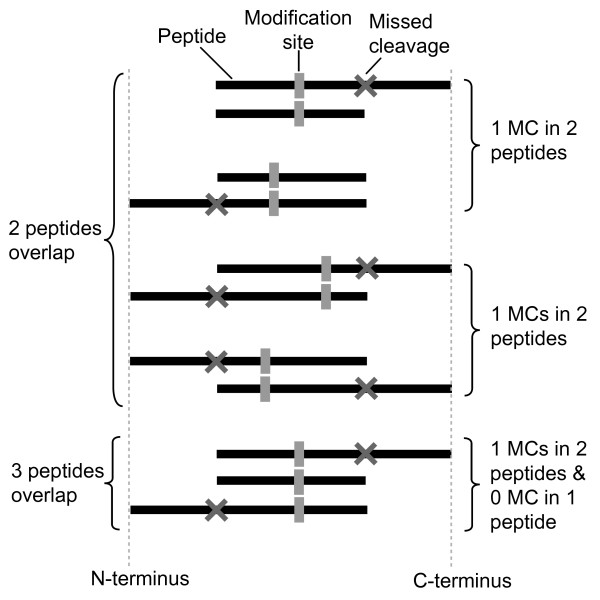
**Filter 2: using two or more overlapping peptides to improve modification confidence**. A peptide can have no missed cleavage or one missed cleavage (MC) at either the N-terminus or C-terminus of the peptide. The modification site has to be found in two or more overlapping peptides for it to be accepted for subsequent analyses. There are a few scenarios where these modification sites can be found. a) There can be one peptide with no missed cleavage overlapping with another peptide with one missed cleavage. b) The modification site can be found in two overlapping peptides each with one missed cleavage. c) In the third scenario, the modification site may be found in three peptides, one peptide with no missed cleavage, and two peptides with one missed cleavage each. In all of the above cases, at least one peptide in the overlapping peptides has to be an unambiguous peptide match. Cases where this was not seen were excluded from all subsequent analyses.

FindMod was used to analyse peptide mass spectra for 2,607 yeast proteins out of a total ~6,500 (representing 40% of the total proteome) for the presence of mono- and di-methylation. A tailor-made mass tolerance was calculated for each spectrum to reduce spurious peptide matches; the average of this for all spectra was ± 0.04 Da. Of all the 24,105 FindMod queries, there were 17,471 matches to potentially methylated peptides (Figure 1). Five filtering strategies, used sequentially, were then applied to this set to find methylation sites of very high confidence. The first filter removed peptides that matched to unmodified peptide sequences as these peptide masses are likely to be unmodified peptides. Conversely, peptides masses that did not match to unmodified peptide sequences are likely to be modified, and these were analysed with the second filter. The second filter removed any peptides that contained D or E residues, as artifactual methylation may result from partial methyl esterification of D or E residues [39]. The third filter was designed to take advantage of redundancy within each FindMod output, by removing one-off or spurious mass spectra. It searched for modifications that were found in two or more overlapping peptides (Figure 2), and took advantage of the reduced efficiency of tryptic cleavage at methylated residues [32], where overlapping peptides with missed cleavages were likely to be found. The fourth filter reduced FindMod false positives by considering whether modifications found by FindMod were unambiguous or ambiguous. An unambiguous modification had only one FindMod match against one query peptide mass (Additional File 1), an ambiguous modification had more than one match against a query mass (Additional File 1). For the peptide to be included in the final set of methylated peptides, at least one peptide in the overlapping peptides had to be an unambiguous peptide match. The use of these 4 filters resulted in 169 high confidence methylated peptides, from 17,471 initial low confidence matches (Figure 1).

While overlapping peptides helped localise methylation sites to one or more peptides, they did not necessarily localise the methylation to one amino acid. To address this, we used a fifth filter. When two or more modified peptides that passed filters 1-4 were also found to overlap and share the same modification site, the modification was classified as high confidence and kept. Note that any results for lysine trimethylation were discarded from the study since it is near-isobaric to lysine acetylation. From this filtering process, we found 40 lysine-methylated proteins with 45 lysine methylation sites: 25 with mono- and 20 with di-methylation. Similarly, we found 31 arginine-methylated proteins with 38 arginine methylation sites: 20 with mono- and 18 with di-methylation. There were 5 proteins that contained both arginine and lysine methylation. The list of high confidence methylated proteins and methylation sites are shown in Table 1, additional information on these high confidence methylated peptides and methylation sites are shown in Additional file 2 and Additional file 3 correspondingly.

**Table 1:** List of methylated proteins and methylation sites discovered by FindMod.

Gene name	Swiss-Prot accession	Description	Methylated residues^a^
ARC40	P38328	Actin-related protein 2/3 complex subunit 1	mK121
ATP2	P00830	ATP synthase subunit beta, mitochondrial precursor	mK196
CDC11	P32458	Cell division control protein 11	dR35
DIP2	Q12220	U3 small nucleolar RNA-associated protein 12	mK350
DNF1	P32660	Probable phospholipid-transporting ATPase DNF1	mK541
ECM29	P38737	Proteasome component ECM29	mR542, dR1112
EDE1	P34216	EH domain-containing and endocytosis protein 1	mR252
EMG1	Q06287	Essential for mitotic growth 1	mK147
ERB1	Q04660	Ribosome biogenesis protein ERB1	mK577, mK581
FKS1	P38631	1,3-beta-glucan synthase component FKS1	mR946, dR946, dR952, mR962, dR962, dR1527
			
GCD10	P41814	tRNA (adenine-N(1)-)-methyltransferase non-catalytic subunit TRM6	mK436, mR447
			
GCN1	P33892	Translational activator GCN1	dK1446
GCN20	P43535	Protein GCN20	dK656
GUS1	P46655	Glutamyl-tRNA synthetase, cytoplasmic	mR371
HAS1	Q03532	ATP-dependent RNA helicase HAS1	dK444
IMD3	P50095	Probable inosine-5'-monophosphate dehydrogenase IMD3	mR168
			
ISW1	P38144	ISWI chromatin-remodeling complex ATPase ISW1	mK14
LEA1	Q08963	U2 small nuclear ribonucleoprotein A'	dR141
MPG1	P41940	Mannose-1-phosphate guanyltransferase	dK299
MRPL17	P36528	54S ribosomal protein L17, mitochondrial precursor	mK70
MRPL20	P22354	54S ribosomal protein L20, mitochondrial precursor	mK104
NIP1	P32497	Eukaryotic translation initiation factor 3 subunit C	mK514
NOC2	P39744	Nucleolar complex protein 2	mK384
NOG2	P53742	Nucleolar GTP-binding protein 2	dR336
NOT1	P25655	General negative regulator of transcription subunit 1	mR256
POL12	P38121	DNA polymerase alpha subunit B	mK84
PRP43	P53131	Pre-mRNA-splicing factor ATP-dependent RNA helicase PRP43	dK662
			
PRT1	P06103	Eukaryotic translation initiation factor 3 subunit B	mR572
PSD2	P53037	Phosphatidylserine decarboxylase proenzyme 2 precursor	mR252
			
PYK1	P00549	Pyruvate kinase 1	mR216, dR216
RPA2	P22138	DNA-directed RNA polymerase I subunit RPA2	dK513
RPB2	P08518	DNA-directed RNA polymerase II subunit RPB2	mR496
RPL16A	P26784	60S ribosomal protein L16-A	dK148
RPL18A, RPL18B	P07279	60S ribosomal protein L18	mR105
RPL1A, RPL1B	P53030	60S ribosomal protein L1	dK207
RPL20A, RPL20B	P0C2I0	60S ribosomal protein L20	dK47
RPL27A, RPL27B	P0C2H6, P0C2H7	60S ribosomal protein L27	dR15, dK133
RPL2A, RPL2B	P05736	60S ribosomal protein L2	dR21
RPL3	P14126	60S ribosomal protein L3	mR275, dK384
RPL4A	P10664	60S ribosomal protein L4-A	dR84, mK104
RPL7A	P05737	60S ribosomal protein L7-A	mR218
RPL7B	Q12213	60S ribosomal protein L7-B	mR218
RPL8B	P29453	60S ribosomal protein L8-B	mK15, mK43, dK241
			
RPN2	P32565	26S proteasome regulatory subunit RPN2	mK376
RPS11A, RPS11B	P26781	40S ribosomal protein S11	mR67
RPS13	P05756	40S ribosomal protein S13	mK140
RPS17A	P02407	40S ribosomal protein S17-A	dK59
RRP5	Q05022	rRNA biogenesis protein RRP5	dR215, dK769
RSC1	P53236	Chromatin structure-remodeling complex subunit RSC1	mR454
RSC30	P38781	Chromatin structure-remodeling complex protein RSC30	mR692
			
RVB2	Q12464	RuvB-like protein 2	mK412
SKI3	P17883	Superkiller protein 3	dK1088
SMB1	P40018	Small nuclear ribonucleoprotein-associated protein B	mK138, mK145
SNF2	P22082	Transcription regulatory protein SNF2	dK1028
SNU114	P36048	114 kDa U5 small nuclear ribonucleoprotein component	dK356, mK935
SSB1	P11484	Heat shock protein SSB1	dR513
SSB2	P40150	Heat shock protein SSB2	dR513
TDH3	P00359	Glyceraldehyde-3-phosphate dehydrogenase 3	dR11
TIF32	P38249	Eukaryotic translation initiation factor 3 subunit A	dK192
TUB2	P02557	Tubulin beta chain	dR318
URA7	P28274	CTP synthase 1	dK28
USO1	P25386	Intracellular protein transport protein USO1	mK119
UTP22	P53254	U3 small nucleolar RNA-associated protein 22	mK1158
VPS52	P39904	Vacuolar protein sorting-associated protein 52	mR224
YKU70	P32807	ATP-dependent DNA helicase II subunit 1	dR549
YPR097W	Q06839	PX domain-containing protein YPR097W	dK249

**a**: m, monomethylated; d, dimethylated

### Confirmation of FindMod protein methylation

To establish the accuracy of our methylation discovery approach, we theoretically digested all known methylated proteins in Swiss-Prot and analysed the resulting peptides with our FindMod approach. We supplemented this with a larger set of theoretically methylated proteins. The average true positive rate for FindMod at 0.04 Da was 89%. For methylation sites in Swiss-Prot, FindMod had a true positive rate of 100% for monomethyl-K, 98% for dimethyl-K, and 76% for dimethyl-R (Table 2a). The true positive rate for monomethyl-R could not be accurately estimated since the number of test cases was insufficient for accurate evaluation. Similarly, the true positive rate for the artificial methylation set was 78% for monomethyl-K, 89% for dimethyl-K, and 90% for both monomethyl-R and dimethyl-R (Table 2b). Additional results for the evaluation of the true positive rate of FindMod are shown in Additional file 4.

**Table 2:** True positive rate at mass tolerance of 0.04 Da.

Type of methylation	True positive rate (%)	No. of methylation sites correctly matched	No. of methylation sites tested	No. of peptides tested
**a) Non-redundant known mono- and di-methylation**				
Monomethyl-K	100	27	466	1,201
Dimethyl-K	98	89	280	699
Monomethyl-R	N.D.^a^	N.D.^a^	3	6
Dimeth-R	76	28	137	495
				
**b) Artificial mono- and di-methylation**				
Monomethyl-K	78	217	11,377	30,782
Dimethyl-K	89	3,673	11,377	30,691
Monomethyl-R	90	453	6,941	18,728
Dimethyl-R	90	2,140	6,902	18,594

**a**: N.D. - not determined

To further assess the accuracy of the FindMod approach, methylation sites discovered by FindMod were cross-referenced with known methylation sites in the literature and databases. Whilst only a small number of proteins are documented as methylated in the literature, we confirmed 3 proteins (Ssb1p, Ssb2p, Tub2p) as methylated (Table 3). If we included methyl-lysine sites in peptides containing D and E, we also confirmed the methylation of Tef1p and Rpl23p. This included 3 lysine methylation sites (K30, K79, and K390) from Tef1p, and 1 lysine dimethylation site (K110) from Rpl23p [14] (Additional file 5). Furthermore, we found 15 methylated ribosomal proteins in *S. cerevisiae*, consistent with the presence of methylation sites in ribosomal proteins of eukaryotes, such as *S. cerevisiae *[12-15,40], *S. pombe *[41], *A. thaliana *[42], and human [43-46].

**Table 3:** Methylated proteins identified independently by both FindMod and described in the literature.

Ordered locus name (Swiss-Prot accession)	Protein name	Literature^a^
YDL229W (P11484)	Ssb1p	Wang and Lazarides [48], Wang *et al. *[47], Iwabata *et al. *[33]
YNL209W (P40150)	Ssb2p	Wang and Lazarides [48], Wang *et al. *[47], Iwabata *et al. *[33]
YPR080W, YBR118W (P02994)	Tef1p/Tef2p/eEF1αp	Cavallius *et al. *[16], Iwabata *et al. *[33]
YBL087C, YER117W (P04451)	Rpl23A/Rpl23B	Porras-Yakushi *et al. *[14]

YFL037W (P02557)	Tub2p	Iwabata *et al. *[33]

**a**: Proteins previously known to be methylated in yeast and in other organisms

### Discovery rate of methylated peptides, unmodified peptides, and lysine and arginine-methylated residues

The discovery rate of a peptide is the frequency of protein identifications in which a particular peptide is observed. Methylated peptides with low discovery rates are likely to be sub-stoichiometric and partially methylated. It was predicted that there should be many more unmodified peptides than methylated peptides, and that methylated peptides will have a lower discovery rate since they are likely to be sub-stoichiometric. The discovery rate of high confidence methylated peptides was found to be significantly lower than that of unmodified peptides (p < 0.0001). The median discovery rate for unmodified peptides was 0.50, and the median value for arginine and lysine methylated peptides was 0.03. To check that the lower discovery rate of methylated residues was not due to differences in peptide ionisation efficiency, we examined if there was a correlation between the discovery rates of methylated and unmodified residues. In the set of results, there were 69 methylated residues for which the corresponding unmodified residues were also seen. The discovery rate of methylated residues was significantly but weakly correlated with the discovery rate of matching unmodified residues (Kendall's τ = 0.22, p < 0.01), consistent with expected. A list of methylated proteins and the methylation sites discovered by FindMod is shown in Table 1. The discovery rate of all high confidence methylated peptides and methylation sites are shown in Additional file 2 and Additional file 3 correspondingly.

### Biological function, sub-cellular localization, abundance and half-life of methylated proteins

Methylated proteins are known to be involved in several pathways, such as translation [11] and RNA processing [49]. To investigate the function of the methylated proteins from yeast, gene ontology (GO) annotations for all yeast methylated proteins from FindMod analysis and Swiss-Prot were compared to non-methylated yeast proteins (Table 4). It was found that a number of biological processes were enriched with very high statistical significance, specifically translation, ribosome biogenesis and assembly, RNA metabolic process, and organelle organization and biogenesis. The molecular function of structural activity, RNA binding, and translation regulator activity were also significantly enriched. As may be expected from the above, methylated proteins were significantly enriched in the cellular components of the ribosome and cytoplasm.

**Table 4:** Methylated proteins from yeast are enriched in specific processes, functions and components.

Rank	Term (GO ID)	n_1,1_^a^	n_1,2_^b^	n_2,1_^c^	n_2,2_^d^	Corrected *p*-value
**Biological process**						
1	Translation (6412)	37	48	305	5712	3.57 e-23
2	Ribosome biogenesis and assembly (42254)	20	65	311	5706	5.03 e-7
3	RNA metabolic process (16070)	21	64	664	5353	0.01
4	Organelle organization and biogenesis (6996)	30	55	1230	4787	0.04
						
**Molecular function**						
1	Structural molecule activity (5198)	31	54	304	5712	3.95 e-17
2	Translation regulator activity (45182)	5	80	47	5969	0.01
3	RNA binding (3723)	10	75	225	5791	0.03
						
**Cellular component**						
1	Ribosome (5840)	33	52	307	5710	5.56 e-19
2	Cytoplasm (5737)	59	26	2701	3316	1.05 e-4

**a**: Number of methylated proteins with this GO slim term

**b**: Number of non-methylated proteins with this GO term

**c**: Number of methylated proteins with other GO slim term

**d**: Number of non-methylated proteins with other GO slim term

**Figure 3:**
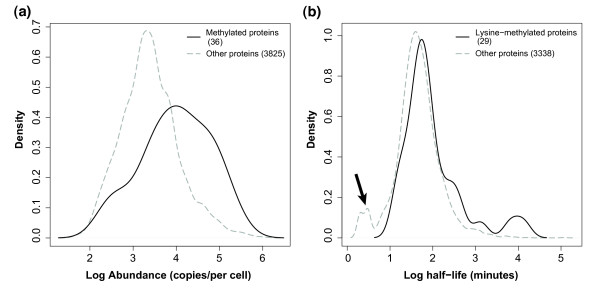
**Distribution of abundance of methylated proteins and half-life of lysine-methylated proteins, versus non-methylated proteins**. **a) **The x-axis represents protein abundance, in copies per cell, as log base 10. The abundance of methylated proteins is represented with a solid line, while the abundance of non-methylated proteins is represented with a dotted line. **b) **The x-axis represents protein half-life, minutes, in log base 10. The half-life of lysine-methylated proteins is represented with a solid line, while the half-life of non-methylated proteins is represented with a dotted line. The arrow points to a group of proteins with very short half-life, seen only in 'other' proteins, which are likely to be unmethylated. Note that in both figures, 'other' proteins are those for which methylation was not found; this group may, however, contain some methylated proteins. Note also that abundance data and half-life data was not available for all yeast proteins in Belle *et al. *(2006) [53] and Ghaemmaghami *et al. *(2003) [50].

Protein abundance data from Ghaemmaghami *et al. *(2003) [50] was used to compare the abundance of methylated proteins to non-methylated proteins. This was used to determine if lower abundance proteins, more likely to be involved in signal transduction and regulation [51], are methylated. Methylated proteins were found to have a higher median abundance of 11500, as compared to non-methylated proteins, which had a median abundance of 2220 (p < 0.0001, Figure 3a). Despite this, several methylated proteins of low abundance were seen including 5 proteins of less than 1000 molecules per cell. These included Snf2p (217 copies/cell), Snu114p (300 copies/cell), Mrpl20p (358 copies/cell), and Rpl3p (450 copies/cell). Examples of proteins with high abundance are Rp1Bp (265,000 copies/cell) and Tdh3p (169,000 copies/cell).

The methylation of lysine residues has been suggested to block their ubiquitination, leading to a longer protein half-life [52]. To investigate this possibility, protein half-life data from Belle *et al. *(2006) [53] was used to compare the half-life of lysine-methylated proteins to non-methylated proteins. Interestingly, we found methylated proteins had a longer median half-life of 66 minutes, as compared to 43 minutes for non-methylated proteins (p = 0.012, Figure 3b). A striking difference between the methylated and non-methylated proteins was the absence of a group of proteins with very short half-life (see arrow in Figure 3b). Despite this, our approach also identified 18 methylated proteins with half-life less than 60 minutes. Examples of methylated proteins with shorter half-lives are Rrp5p (15 minutes), Ski3p (32 minutes), and Snu114p (52 minutes). Examples of proteins with long half-life are Utp22p (13,266 minutes) and Atp2p (6,627 minutes), although we note that these numbers may be erroneous estimations in the Belle *et al. *study (2006) [53]. Although the abundance and half-lives of methylated proteins could be analysed more precisely by comparing methylated proteins to other proteins from the same GO slim biological process, this approach was limited by the relatively small number of methylated proteins (66 proteins) in the dataset. Methylated proteins mapped to 33 gene ontology biological process categories, with an average of 2 proteins per category, which was unsuitable for appropriate statistical analyses.

### Interplay of methylation and other post-translational modifications

To see if lysine methylation might block ubiquitination, the Ubipred software [54] was used to predict if known methylated lysine sites are also subject to ubiquitination. The Ubipred software has an accuracy of 84.4% and is thus sufficiently reliable for this test. It was found that 43% of high-confidence lysine methylation sites were also predicted to be ubiquitination sites. This result lends support to the hypothesis that methylation might block ubiquitination, potentially prolonging the half-life of lysine-methylated proteins.

It has recently been reported that the methylation of arginine can regulate the phosphorylation (or dephosphorylation) of some proteins [55-61]. To investigate whether there is evidence of interplay between arginine methylation and phosphorylation in *S. cerevisiae*, we examined the proportion of arginine-methylated proteins that are known to be phosphorylated in databases and in the literature. It was found that 94% (30/32) of arginine-methylated yeast proteins are known to be phosphorylated. This is a considerable increase over the 38% (2,548/6,709) of all *S. cerevisiae *proteins known to be phosphorylated and suggests a possible interplay of arginine methylation and phosphorylation [55-61].

### Arginine and lysine methylation motifs

To determine if methylation sites are enriched in specific sequence-motifs, all yeast methylation sites from FindMod analysis and the Swiss-Prot database were analysed to find enriched sequence-motifs. Methionine was found to be at position -1 from lysine methylation in 5 FindMod sites and two additional methylation sites previously documented in *S. cerevisiae *(Table 5). This presence of methylation was of very high statistical significance (p = 1.18 × 10^-6^) as compared to that expected in any random sequence of yeast proteins. By contrast, residues found to be significantly enriched adjacent to arginine methylation included W at position -4 (p = 1.50 × 10^-7^), and G at position -3 (p = 6.08 × 10^-6^). While it was previously known that arginine methylation is found in RGG motifs, Wooderchak *et al. *(2008) [62] showed that arginine methylation is also found in RXG and RGX motifs. No known *S. cerevisiae *methylation sites documented in Swiss-Prot contained the **R**GG, **R**GX, or **R**XG-motifs. However, FindMod found 7 methylation sites with the **R**XG or **R**GX motifs. Two methylation sites, Tdh3p dimethyl-R11 and Rpl4Ap dimethyl-R84, matched to the GXX**R**XG motif, which conforms with the known **R**XG motif and the additional GXX**R **motif found in this study. Three methylation sites had the novel WXXX**R **motif.

**Table 5:** Lysine and arginine methylation motifs.

Gene name	Swiss-Prot accession	Methylation site^a^	Motif^b^
**MK motif**			
Gcd10p	P41814	meth-K436^2^	RGKLHPLMTM**K**GGGGYLMWCH
Pfk2p	P16862	meth-K180^1^	HSYTDLAYRM**K**TTDTYPSLPK
Rpl23Ap	P04451	dimeth-K110^1^	GVIANPKGEM**K**GSAITGPVGK
Rps17Ap	P02407	dimeth-K59^2^	KIAGYTTHLM**K**RIQKGPVRGI
Rvb2p	Q12464	meth-K412^2^	LISVAQQIAM**K**RKNNTVEVED
Ura7p	P28274	dimeth-K28^2^	VLASSTGMLM**K**TLGLKVTSIK
Uso1p	P25386	meth-K119^2^	NGKYPSPLVM**K**QEKEQVDQFS
			
**RGx or RxG motif**			
Ecm29p	P38737	dimeth-R1112^2^	LAKSSALWSS**R**KGIAFGLGAI
Gus1p	P46655	dimeth-R371^2^	IYRCNLTPHH**R**TGSTWKIYPT
Rpl27Bp	P38706	dimeth-R15^2^	LKAGKVAVVV**R**GRYAGKKVVI
Rpl4Ap	P10664	dimeth-R84^2^	IPRVGGGGTG**R**SGQGAFGNMC
Rps11Bp	P26781	dimeth-R67^2^	KCPFTGLVSI**R**GKILTGTVVS
Tdh3p	P00359	dimeth-R11^2^	MVRVAINGFG**R**IGRLVMRIAL
Tub2p	P02557	dimeth-R318^2^	GRYLTVAAFF**R**GKVSVKEVED
			
**WxxxR and/or GxxR motif**			
Cdc11p	P32458	dimeth-R35^2^	VMIVGQSGSG**R**STFINTLCGQ
Ecm29p	P38737	meth-R542^2^	ARLFNIWGTV**R**TNRFDIIEES
Fks1p	P38631	meth-R946^2^, dimeth-R946^2^	TLRTRIWASL**R**SQTLYRTISG
Fks1p	P38631	dimeth-R1527^2^	YHRNSWIGYV**R**MSRARITGFK
Rpl4Ap	P10664	dimeth-R84^2^	IPRVGGGGTG**R**SGQGAFGNMC
Rpl7Ap, Rpl7Bp	P05737	meth-R218^2^	SNPSGGWGVP**R**KFKHFIQGGS
Rsc30p	P38781	meth-R692^2^	SIKSFSSGNN**R**FHSNGKEFLF
Tdh3p	P00359	dimeth-R11^2^	MVRVAINGFG**R**IGRLVMRIAL

**a**: Evidence for the presence of the methylation site on this protein 1: Swiss-Prot, 2: methylation site confirmed by FindMod analysis

**b**: Methylation site matching the specified motif is underlined, the methylation site is highlighted in **bold**.

## Discussion

### Large-scale discovery of lysine and arginine methylation sites

In this study, 45 lysine methylation sites and 38 arginine methylation sites were identified in 66 proteins in the *S. cerevisiae *proteome. These include 4 proteins previously known to be methylated in yeast or in other organisms and 15 proteins that are functionally related to others known to be methylated. Our findings support earlier studies [31-33] that suggested methylation to be quite widespread. Whilst many of our methylation sites are novel and have not been confirmed by MS-MS, the filters and replicate analyses we used in association with the FindMod tool provided a robust means by which protein methylation could be detected. The false positive rate was estimated to be 11% at 0.04 Da mass error. Notwithstanding this, it should be noted that whilst we did study 2,607 proteins from yeast, this is only ~40% of the total yeast proteome. Therefore, we expect that up to 60% of methylated proteins would have been missed. Further methylation sites may have been missed due to difficulties in mass spectrometric detection; an example is methylarginine, which is often found in arginine- and glycine-rich regions that produce tryptic peptides that are too small for routine MALDI-TOF analysis.

### Discovery rates may reflect the sub-stoichiometric nature of methylation

Previous research has highlighted that methylated peptides are difficult to discover [32] and this is made more difficult because methylation is sub-stoichiometric [34]. For example, sub-stoichiometric levels of methylations were observed in the human heterogeneous nuclear ribonucleoprotein K (hnRNP K), in which < 33% of hnRNP K were asymmetrically dimethylated at R303, and < 10% were monomethylated at R287 [56]. Our results from FindMod analysis support these observations since the proportion of methylated peptides seen for any protein was very low. The sub-stoichiometric nature of methylation events was also supported by a weak but significant correlation between the discovery rates of modified and unmodified paired peptides. However, there may be explanations, other than biological, for the lower discovery rate of modified peptides. These included inefficient trypsin cleavage which occurs C-terminal to methylated lysine and arginine residues [32] and differences in MALDI-ToF ionisation of the methylated peptides as seen with different proteotypic peptides [63].

### Methylated proteins are involved in specific biological functions and processes, are higher in abundance and have longer half-life

Methylated proteins were found to be enriched for specific biological processes, molecular functions and sub-cellular localizations. Firstly, methylated proteins were enriched in translation, ribosome biogenesis and assembly. This is consistent with previous studies in which methylated proteins have been linked to translation in *Escherichia coli*, *S. cerevisiae*, and *Schizosaccharomyces pombe *[11]. Ribosomal proteins are also known to show lysine or arginine methylation, for example the ribosomal proteins L10a, L12, and L26a of *Arabidopsis *[42]. Secondly, the methylated proteins described here were found to be involved in RNA metabolic processes and are involved in RNA binding. This is consistent with the function of several proteins known to be methylated at RG-rich motifs [49]. The methylation of arginine in RG-rich motifs is conserved in human, and their RNA binding activity is also conserved [32]. One such example is the fragile X mental retardation protein (FMRP) [25]. Thirdly, our methylated proteins were enriched in the ribosome and the cytoplasm. This is consistent with the sites of translation and association with RNA inside the cell [22,23]. Whilst the lack of methylated proteins enriched in the nucleus and nucleolus was not expected, these may have arisen due to our reduced set of proteins for analysis (40% of the yeast proteome). In addition, nuclear proteins such as histone and Npl3p are known to have peptides with multiple modification sites but these were not searched for in this study. Methylated proteins found in this study were significantly higher in abundance than proteins currently known to be non-methylated. This is partly explained by ribosomal proteins and proteins involved in translation, some of which we found to be methylated, being of very high abundance [50,64]. Methylated proteins were also found to be of longer average half-life. This may be due to their role in translation [11], where ribosomal proteins are generally stable [53].

### Interplay of methylation and other post-translational modifications

The methylation of lysine is known to block the action of ubiquitin ligase [65], preventing proteins from degradation via the ubiquitin/proteasome system [52,66]. Our observation of a distinct group of low half-life proteins in *S. cerevisiae*, none of which were methylated, suggests that lysine methylation might be on many proteins and prevent their ubiquitination. The limited number of ubiquitination sites currently known on yeast proteins [67,68] makes it currently difficult to check if lysine methylation, as found in this study, is found on residues that can also be poly-ubiquitinated. However, our prediction of putative ubiquitination sites [54] showed that 43% of the lysine methylation sites in 40 proteins may be ubiquitinated.

Several studies suggested that there is interplay between arginine methylation and phosphorylation of some proteins [55-61]. Arginine methylation may antagonise phosphorylation [56,57], act as a switch to enable the binding of phosphatase to encourage dephosphorylation [60], or encourage phosphorylation [59]. On the other hand, phosphorylation can either interfere with arginine methylation [58,61], or promote the recruitment of arginine methyltransferase [55]. We found that the majority of arginine-methylated proteins in our study (30 out of 32 or 94%) are known from the literature to be phosphorylated, suggesting an interplay between arginine methylation and phosphorylation in these proteins. However these arginine methylation and phosphorylation sites were not necessarily directly adjacent in the protein sequence.

### Arginine and lysine methylation motifs

Motif analysis showed that many methylation sites described here conform with previously known motifs. For example, 7 arginine methylation sites discovered by FindMod conformed with the known **R**XG and **R**GX motifs [62]. Arginine methylation sites were also enriched in GXX**R **motifs, which correlated with the enrichment of glycine residues nearby arginine methylation sites [69]. In addition, two experimentally verified methylated sites in Pfk2p and Rpl23Ap annotated in Swiss-Prot along with 5 FindMod sites suggests the existence of a M**K **lysine methylation motif. The discovery of the novel enriched methylation motif WXXX**R **supports the possibility that there are more methylation sites to be found in *S. cerevisiae*. These also raise an interesting question concerning which motifs are methylated by specific methyltransferases. Methyltransferases responsible for most methylation sites are also unknown (e.g. Tef1p K30, Pfk2p K180), and the function of several methyltransferase proteins in *S. cerevisiae *remain poorly characterized [13]. Therefore, more experiments are required to elucidate the function of methylation in *S. cerevisiae*.

## Conclusions

This study is a step towards the definition of the methyl proteome of *S. cerevisiae*. It will be useful to guide future experiments on its predominance and role in the cell. For example, experiments are needed to elucidate the function of methylation and how each site is regulated, which with the exception of histone methylation is largely unknown. Secondly, experiments to investigate whether methylation sites overlap with poly-ubiquitination sites, and therefore prevent protein degradation via the ubiquitin/proteasome pathway could be undertaken. Thirdly, it will be important to understand whether the functions of methylated proteins are co-regulated by ubiquitination, phosphorylation or other post-translational modifications. Finally, the ultimate goal in studying methylation should be to build networks of methylated proteins, their interaction partners and modifying enzymes to elucidate their dynamics as a system, similar to previous work on protein phosphorylation [70-72].

## Methods

### MALDI-ToF mass spectra for *S. cerevisiae*

This study employed MALDI-ToF peptide mass fingerprinting spectra from the large-scale characterization of protein complexes in *S. cerevisiae *[38]. There were 36,854 peptide mass spectra containing 1.2 million empirical masses, with an average mass error of 0.02 Da. These were from 2,607 proteins out of ~6,500 proteins (40%) in the yeast proteome, whereby each protein had an average of 11 spectra or at least 3 spectra. Peptide masses corresponding to unmodified peptides or tryptic peptides of porcine trypsin were removed, as were peptides less than 500 Da.

### Tailor-made mass tolerance for each empirical spectrum

An error threshold was calculated for each of the 36,854 spectra; this was possible as the identity of all proteins was known. For each spectrum, the mass differences between the empirical and theoretical mass of all known unmodified peptides were calculated. The average and median mass tolerance was 0.04 Da. To ensure high accuracy of methylation discovery, only spectra with a mass error (Additional file 6) that was lower than 0.1 Da were used for the identification of methylation sites.

### FindMod analysis of yeast proteins

Each peptide mass spectra was analysed with FindMod [35]. A bulk submission web interface to FindMod was developed http://ca.expasy.org/tools/findmod/findmod_batch.html. Each FindMod query used the UniProt accession number for the protein identified through peptide mass fingerprinting (from Gavin *et al.*, 2006) [38], the experimental peptide masses for this protein and the tailor-made mass tolerance in Da. Other FindMod parameters included the use of monoisotopic mass, a maximum of 1 missed cleavage by trypsin, no amino acid substitutions, that the peptides were M+H^+ ^and could contain oxidised methionine or tryptophan. The peptide masses were matched to theoretical peptides generated from the precursor sequence. The program searched for 71 types of post-translational modifications in all experimental peptide masses [35], including mono-, di-, and tri-methylation http://www.expasy.ch/tools/findmod/findmod_masses.html. Matches to 6 types of modifications were removed from the analyses, as they are not found in *S. cerevisiae *or may lead to many false positives due to their low mass; for more details see Additional file 6. The Swiss-Prot database version 51.6 and TrEMBL version 34.6 [73] were used for the FindMod matches.

### Filters to remove low quality methylation sites

For the methylated peptides to be included in the analysis, they needed to pass the following 5 filters. The peptides 1) cannot be an unmodified peptide, 2) had to contain no Asp or Glu residues, and 3) have no or one missed tryptic cleavage. In addition, 4) the peptide must have two or more overlapping peptides and at least one peptide in the overlapping peptides had to be an unambiguous peptide match. 5) When two or more modified peptides that passed filters 1-4 were also found to overlap and share the same modification site, the modification was classified as high confidence and kept. The use of overlapping peptides to improve the reliability of methylation site is facilitated by methylation sites found at the C-terminus of peptides. Trypsin cleavage at methylated arginine and lysine has been observed in many LC-MS/MS experiments [32,74-77], and is less efficient than at non-methylated residues. A list of tryptic peptides with C-terminal methylated amino acids, identified by LC-MS-MS, is shown in Additional file 7.

### Calculation of discovery rate

The discovery rate for an unmodified peptide was calculated as the fraction of protein identifications in which the unmodified peptide is observed. In the case of duplicated genes, the counts of protein identifications were summed together because peptide mass fingerprinting cannot distinguish between proteins that do not differ in primary sequence. The discovery rate for a particular unmodified residue in the protein was calculated as the sum of the discovery rate of all the unmodified peptides that contain the residue. Discovery rates were also calculated for modified methylated peptides and methylated residues using the method as described above. Partially methylated peptides are likely to have a low discovery rate. While mass spectra with a maximum mass tolerance of 0.1 Da were used for finding the methylation sites to limit the false positive rate, all available mass spectra with a mass tolerance of up to 1.5 Da were used for the calculation of discovery rate. That is because more mass spectra were needed to increase the sample size for discovery rate calculation.

### Evaluation of the true positive and false positive rate

Swiss-Prot entries with known lysine and arginine methylation sites were obtained from Swiss-Shop http://au.expasy.org/swiss-shop/, for Swiss-Prot release 57.2 [73], by searching the MOD_RES field using the keywords 'methyllysine' and 'methylarginine'. *S. cerevisiae *proteins sequences were downloaded from Swiss-Prot by using the query 'organism:4932'. The annotation of known methylation sites were obtained from the MOD_RES field of the Swiss-Prot entry, and type of methylation were determined from the standard RESID nomenclature [78]. The proteins were processed into mature forms where appropriate; these contain no signal peptides, propeptides, intein regions, and only consists of protein chains annotated by the 'CHAIN' field of the Swiss-Prot entry. For each M or W residues in a peptide, the mass of methionine and tryptophan oxidation was added to the total mass of the peptide. Only methylated peptides with a maximum of one-missed cleavage and with masses between 500 and 3,000 Da were used. Since lysine trimethylation is near-isobaric to lysine acetylation, trimethylation was not included in the analysis. Two *in silico *test sets, the known methylation set and the artificial methylation set, were used to evaluate the true positive rate of FindMod for the discovery of mono- and di-methylation on arginine and lysine residues. The known methylation test set contained known lysine and arginine methylation sites from Swiss-Prot. The set of sequences from which methylation sites were found was non-redundant at the 90% identity level, generated using UniRef90 [79]. This test set included 883 known mono- and di-methylation sites. The artificial mono- and di-methylation sites on lysine and arginine residues were generated by simulated methylation on theoretical unmodified peptides. The artificial test set has more data than the known methylation test set, to allow more accurate estimation of the true positive rate. Approximately 6% of lysine residues from *S. cerevisiae *protein sequences were randomly sampled to generate artificially methylated peptides for monomethyl-K. The sampling procedure was repeated for dimethyl-K, monomethyl-R, and dimethyl-R. The second test set was referred to as the artificial methylation set, and contained 36,594 artificial mono- and di-methylation sites.

The true positive rate of FindMod, with the 5 filters described above, was evaluated using known methylation sites and artificial methylation sites. Removal of peptides containing D or E residues were not required since no artifactual methylation on D or E residues were introduced to the *in silico *test sets. The true positive rate was evaluated at the mass tolerance of 0.04 Da, since this was the median mass tolerance all empirical for peptide masses [38]. For each test set, a true positive FindMod match requires the residue, sequence position and the type of methylation to be correctly matched. The true positive rate of FindMod, was calculated as the number of true positive matches divided by the sum of the number of false positive matches and the number of true positives, represented as a percentage.

### Arginine and lysine methylation motif analysis

Ten amino acid residues N-terminal and C-terminal to each methylation site were included in the motif analysis. The number of times each amino acid occurs at each of these positions was counted. For any methylation site less than 10 residues from the N- or C-terminus of the protein, positions beyond the limit of the sequence were disregarded. To measure whether an amino acid was significantly enriched at each position, a p-value was calculated using the *prop.test *function in the R statistical package. A one-sided statistical test was used, with an alternative hypothesis that there was an enrichment of amino acid frequency over the average frequency. Bonferroni's correction was used to correct the p-value calculated by *prop.test *to reduce false positives.

### Functional analysis and statistical tests

Functional data co-analysed with modifications were protein abundance [50], protein half-life [53], Gene Ontology (GO) slim (from *Saccharomyces *Genome Database, ftp://ftp.yeastgenome.org/yeast/) [80] and protein complexes [81]. Nonparametric tests were used for all statistical analyses. Protein abundance data, in copies per cell, was from Ghaemmaghammi *et al. *(2003) [50]. Protein half-life data, in minutes, was from Belle *et al. *(2006) [53]. To investigate if lysine methylation might block ubiquitination, the Ubipred software [54] was used to predict if known methylated lysine sites are also subject to ubiquitination. To investigate if arginine methylated proteins were co-regulated by phosphorylation, the Swiss-Prot database release 57.2 [73] was examined to see if methylated proteins also had experimentally determined protein threonine, serine, and tyrosine phosphorylation sites. Mann-Whitney tests, a non-parametric substitute for Student's t-test, were used to compare between two samples. Kendall's correlation coefficient, a non-parametric substitute for Pearson's correlation coefficient, was used to measure the significance of the correlation between two samples. GO slim term enrichment was assessed using Fisher's exact test and Bonferroni correction [82]. All statistical analyses were performed using the R statistical package version 2.2.1 [83].

## Abbreviations

Da: Daltons; GO: gene ontology; LC-MS/MS: liquid chromatography tandem mass spectrometry; MALDI-ToF: Matrix assisted laser desorption ionisation - time of flight; PTM: post-translational modification.

## Authors' contributions

CNIP designed the method for searching arginine and lysine methylation sites using FindMod, performed the statistical and bioinformatics analyses, and wrote the manuscript. EG implemented the FindMod bulk submission program. MRW supervised the project and critically reviewed the manuscript. All authors read and approved the manuscript.

## Supplementary Material

Additional file 1 **Examples of ambiguous and unambiguous peptide matches**. This file contains examples of ambiguous and unambiguous peptide matches. Click here for file

Additional file 2 **List of lysine- and arginine-methylated peptides**. This file contains the list of all high confidence arginine- and lysine-methylated peptides, and their corresponding discovery rates. Click here for file

Additional file 3 **List of lysine and arginine methylation sites**. This file contains the list of all high confidence methylated residues and their corresponding discovery rates. Click here for file

Additional file 4 **Additional benchmarking results**. Evaluation of the true positive rates of FindMod using different range of mass tolerance from 0.01 to 0.10 Da, and the known non-redundant methylation test set and the artificial methylation test set. Click here for file

Additional file 5 **List of methylated peptides of Tef1p and Rpl23p discovered by FindMod**. This file contains the list of methylated peptides of Tef1p and Rpl23p found by FindMod. These peptides may contain E and D residues, as E and D residues were not filtered for the analysis. Click here for file

Additional file 6 **Supplementary methods**. This file describes how the tailor-made error tolerances was calculated, and also provide a list of low-quality post-translational modifications that were excluded from FindMod's analysis. Click here for file

Additional file 7 **List of methylation at C-terminus of peptides**. This file is a list of peptides with methylation at C-terminus of peptides, collected from literature. Click here for file
