# Supplementary material for: Identification of arginine- and lysine-methylation in the proteome of Saccharomyces cerevisiae and its functional implications
Source: BMC Genomics. 2010 Feb 5;11:92. doi: 10.1186/1471-2164-11-92 (PMC2830191; doi:10.1186/1471-2164-11-92)
Supplement: Additional file 1 — Examples of ambiguous and unambiguous peptide matches. This file contains examples of ambiguous and unambiguous peptide matches. [file 1471-2164-11-92-S1.DOC]

## *Additional File 1: Supplementary Table 1 – Examples of unambiguous and ambiguous modified peptide matches for elongation factor 1-α*

An example of overlapping peptide is shown in Table S1a, where both peptides share residues 21 to 30. The first peptide has a methylation unambiguously predicted to be at K30, while the modification sites in the second peptide is ambiguous and could either be K30 or K36. Using overlapping peptides help resolves this ambiguity and assigns the methylation site to residue 30.

| **a) Two input masses, each with one unambiguous modified peptide match** | | | | | |  |  |
| --- | --- | --- | --- | --- | --- | --- | --- |
| **Query mass** | **Theoretical peptide mass** | **Difference in mass ± tolerance (Da)** | **Additional mass for PTM (Da)^a^** | **PTM type** | **Start** | **Peptide sequence^b^** | **End** |
| 1133.63 | 1119.59 | 14.04 ± 0.046 | 14.02 | Methylation | 21 | STTTGHLIY**K** | 30 |
| 1720.87 | 1706.87 | 14.00 ± 0.043 | 14.02 | Methylation | 21 | STTTGHLIY**K**CGGID**K** | 36 |
|  |  |  |  |  |  |  |  |
| **b) One input mass corresponds to three ambiguous modified peptide matches** | | | | | | | |
| **Query mass** | **Theoretical peptide mass** | **Difference in mass ± tolerance (Da)** | **Additional mass for PTM (Da)^a^** | **PTM type** | **Start** | **Peptide sequence^b^** | **End** |
| 1161.60 | 1119.59 | 42.01 ± 0.029 | 42.01 | Acetylation | 21 | STTTGHLIY**K** | 30 |
| 1161.60 | 1119.59 | 42.01 ± 0.029 | 42.05 | Trimethylation | 21 | STTTGHLIY**K** | 30 |
| 1161.60 | 1147.66 | 13.94 ± 0.029 | 14.02 | Methylation | 391 | FL**K**SGDAALV**K** | 401 |

a: The additional mass of a modified peptide compared to the mass of the corresponding unmodified peptide.

b: The characters in bold highlights one or more amino acids which can be modified by the corresponding type of PTM.
