# Supplementary material for: Identification of arginine- and lysine-methylation in the proteome of Saccharomyces cerevisiae and its functional implications
Source: BMC Genomics. 2010 Feb 5;11:92. doi: 10.1186/1471-2164-11-92 (PMC2830191; doi:10.1186/1471-2164-11-92)
Supplement: Additional file 4 — Additional benchmarking results. Evaluation of the true positive rates of FindMod using different range of mass tolerance from 0.01 to 0.10 Da, and the known non-redundant methylation test set and the artificial methylation test set. [file 1471-2164-11-92-S4.DOC]

**Additional File 4 - Theoretical test sets to evaluate the true positive rate of FindMod**

To evaluate the effect of changing the mass tolerance on the true positive rate of FindMod, it was evaluated using several mass thresholds between 0.01 and 0.10 Da (Figure 1). In general, dimethylated peptides had greater true positive rates than mono-methylated peptides. For non-redundant known monomethylated lysine, the true positive rate remains at 100% due to the low number of test cases. The true positive rate for monomethylated arginine was not determined. For the artificial monomethylation set, the true positive rate drops steadily from 90% to 81% between 0.01 to 0.1 Da. For the artificial dimethylation set, the true positive rate drops steadily from 92% to 87%. Therefore, to control the reliability of methylation sites found by FindMod, it was determined that the maximum mass tolerance used for the discovery of methylation sites was 0.1 Da.


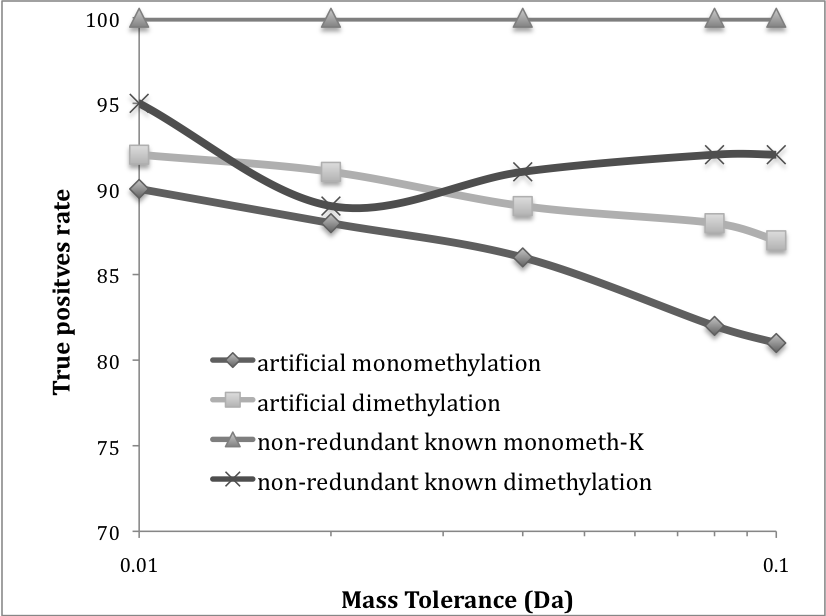


**Figure 1. The true positive rate of FindMod measured at mass tolerance threshold between 0.01 and 0.1 Da.** The vertical axis represents the true positive rate as a percentage, and the horizontal axis represents the mass tolerance (Da) in logarithmic scale. True positive rate was measured for mono- and di- methylated peptides from the non-redundant known methylated peptides set and the artificially methylated peptides set. Artificial monomethylation; diamond with black line, artificial dimethylation; square with grey line, non-redundant known lysine-monomethylation; triangle with grey line, non-redundant known dimethylation; x mark with black line.
