# Supplementary material for: Identification of arginine- and lysine-methylation in the proteome of Saccharomyces cerevisiae and its functional implications
Source: BMC Genomics. 2010 Feb 5;11:92. doi: 10.1186/1471-2164-11-92 (PMC2830191; doi:10.1186/1471-2164-11-92)
Supplement: Additional file 5 — List of methylated peptides of Tef1p and Rpl23p discovered by FindMod. This file contains the list of methylated peptides of Tef1p and Rpl23p found by FindMod. These peptides may contain E and D residues, as E and D residues were not filtered for the analysis. [file 1471-2164-11-92-S5.DOC]

## Additional file 5: Supplementary Table 4 - Methylation sites found by FindMod and supported by literature

| **Gene name** | **Methylation type^a^** | **Modified Residue** | **Peptide Start** | **Sequence** | **Peptide End** | **No. of times seen^b^** | **Evidence^c^** |
| --- | --- | --- | --- | --- | --- | --- | --- |
| Tef1p | Mono | K30 | 6 | SHINVVVIGHVDSGKSTTTGHLIYK | 30 | 11 | 1 |
|  | Mono | K30 | 21 | STTTGHLIYK | 30 | 9 | 1 |
|  | Mono | K30 | 21 | STTTGHLIYKCGGIDK | 36 | 3 | 1 |
|  | Tri | K79 | 70 | GITIDIALWKFETPK | 84 | 1 | 1 |
|  | Tri | K79 | 70 | GITIDIALWK | 79 | 1 | 1 |
|  | Mono | K390 | 384 | KLEDHPK | 390 | 2 | 1 |
|  | Mono | K390 | 385 | LEDHPKFLK | 393 | 4 | 1 |
| Rpl23p | Dimeth | K110 | 107 | GEMKGSAITGPVGK | 120 | 1 | 2 |

**a:** Type and extent of methylation. Mono: monomethylation, Di: dimethylation, Tri: Trimethylation

**b:** Number of times the methylated peptide has been identified

**c:** External evidence for the presence of the methylation site 1: Swiss-Prot, 2: Porras-Yakushi, TR, Whitelegge, JP & Clarke, S: Yeast ribosomal/cytochrome c SET domain methyltransferase subfamily: identification of Rpl23ab methylation sites and recognition motifs. J Biol Chem 2007, 282, 12368-76.
