# Supplementary material for: Identification of arginine- and lysine-methylation in the proteome of Saccharomyces cerevisiae and its functional implications
Source: BMC Genomics. 2010 Feb 5;11:92. doi: 10.1186/1471-2164-11-92 (PMC2830191; doi:10.1186/1471-2164-11-92)
Supplement: Additional file 6 — Supplementary methods. This file describes how the tailor-made error tolerances was calculated, and also provide a list of low-quality post-translational modifications that were excluded from FindMod's analysis. [file 1471-2164-11-92-S6.DOC]

### Additional File 6: Supplementary Methods

### Tailor-made mass tolerance for each empirical spectrum

An error threshold was calculated for each of the 36,854 spectra. This was possible as the identity of all proteins was known. For each spectrum, the mass difference between the empirical and theoretical mass of all known unmodified peptides were calculated. If greater than 10% of the mass differences between the empirical and theoretical mass of peptides were greater than 1.00 Da, then the maximum error found for a peptide would be used. Otherwise, the maximum mass tolerance that was lower than 1.00 Da in the sample was used. The average and median mass tolerance was 0.04 Da. To ensure high accuracy of methylation discovery, only spectra with a tailor made mass error that was lower than 0.10 Da was used for the identification of methylation sites.

### Removing low-quality FindMod peptide matches

After all the peptide mass has been searched with FindMod the results were filtered to remove any low quality results from subsequent analyses. Some peptides mass matches ambiguously to both an unmodified peptide and a modified peptide, these matches are unreliable and are removed from all subsequent analyses. Matches to several types of modifications are also removed from all subsequent analyses. Matches to sulfation were removed because none have been found in yeast (Moore 2003), cis-14-hydroxy-10,13-dioxo-7-heptadecenoic acid aspartate ester is removed since it is only found in plants and n-octanoate because it is only found in mammals (Wilkins et al. 1999). Cysteine sulfinic acid has one known occurrence in yeast, on an active site cysteine residue (Wilson et al. 2004). This modification is removed because we expect very few of these in yeast, and similar argument is made for cysteine sulfenic acid modification. Citrullination has a low mass of (0.984 Da), which could lead to many unspecific matches to this modification, and is also removed from the rest of the analyses.
